# Alcohol consumption and cognitive performance: a Mendelian randomization study

**DOI:** 10.1111/add.12568

**Published:** 2014-07-01

**Authors:** Meena Kumari, Michael V Holmes, Caroline E Dale, Jaroslav A Hubacek, Tom M Palmer, Hynek Pikhart, Anne Peasey, Annie Britton, Pia Horvat, Ruzena Kubinova, Sofia Malyutina, Andrzej Pajak, Abdonas Tamosiunas, Aparna Shankar, Archana Singh-Manoux, Mikhail Voevoda, Mika Kivimaki, Aroon D Hingorani, Michael G Marmot, Juan P Casas, Martin Bobak

**Affiliations:** 1 Department of Epidemiology and Public Health, University College LondonLondon, UK; 2 ISER, University of EssexColchester, UK; 3 Department of Surgery, Division of Transplantation and Clinical Epidemiology Unit, Center for Clinical Epidemiology and Biostatistics, Perelman School of Medicine, University of PennsylvaniaPhiladelphia, PA, USA; 4 London School of Hygiene and Tropical MedicineLondon, UK; 5 Centre for Experimental Medicine, Institute for Clinical and Experimental MedicinePrague, Czech Republic; 6 Division of Health Sciences, Warwick Medical School, University of WarwickCoventry, UK; 7 National Institute of Public HealthPrague, Czech Republic; 8 Institute of Internal Medicine, Russian Academy of Medical ScienceNovobrisk, Russia; 9 Novosibirsk State UniversityNovosibirsk, Russia; 10 Department of Epidemiology and Population Studies, Jagiellonian University Medical CollegeKrakow, Poland; 11 Institute of CardiologyKaunas, Lithuania; 12 INSERM, U1018, AP-HPVillejuif, France; 13 Institute of Cardiovascular Science, University College LondonLondon, UK

**Keywords:** ADH1B, alcohol intake, cognition, memory, processing speed, verbal fluency

## Abstract

**Aims:**

To use Mendelian randomization to assess whether alcohol intake was causally associated with cognitive function.

**Design:**

Mendelian randomization using a genetic variant related to alcohol intake (ADH1B rs1229984) was used to obtain unbiased estimates of the association between alcohol intake and cognitive performance.

**Setting:**

Europe.

**Participants:**

More than 34 000 adults.

**Measurements:**

Any versus no alcohol intake and units of intake in the previous week was measured by questionnaire. Cognitive function was assessed in terms of immediate and delayed word recall, verbal fluency and processing speed.

**Findings:**

Having consumed any versus no alcohol was associated with higher scores by 0.17 standard deviations (SD) [95% confidence interval (CI) = 0.15, 0.20] for immediate recall, 0.17 SD (95% CI = 0.14, 0.19) for delayed recall, 0.17 SD (95% CI = 0.14, 0.19) for verbal fluency and 0.12 SD (95% CI = 0.09, 0.15) for processing speed. The minor allele of rs1229984 was associated with reduced odds of consuming any alcohol (odds ratio = 0.87; 95% CI = 0.80, 0.95; *P* = 0.001; *R*^2^ = 0.1%; *F*-statistic = 47). In Mendelian randomization analysis, the minor allele was not associated with any cognitive test score, and instrumental variable analysis suggested no causal association between alcohol consumption and cognition: −0.74 SD (95% CI = −1.88, 0.41) for immediate recall, −1.09 SD (95% CI = −2.38, 0.21) for delayed recall, −0.63 SD (95% CI = −1.78, 0.53) for verbal fluency and −0.16 SD (95% CI = −1.29, 0.97) for processing speed.

**Conclusions:**

The Mendelian randomization analysis did not provide strong evidence of a causal association between alcohol consumption and cognitive ability.

## Introduction

Numerous observational cross-sectional and longitudinal studies have examined the relationship of drinking alcohol with cognition 1–4. The findings suggest that light-to-moderate drinking reduces the risk of all forms of dementia and is associated with higher cognition test scores. However, observational studies are susceptible to reverse causality or confounding biases, and therefore may not be well suited to elucidate the true effects of alcohol intake. For example, drinkers with alcohol-related health problems, such as impaired cognition, may stop drinking; in this case, non-drinkers would appear to have lower cognitive scores than current drinkers.

Mendelian randomization takes advantage of the properties of genetic variants present from birth and allocated at random according to Mendel's second law 5–8. Gene variants associated with alcohol intake can be used to evaluate the potentially unbiased effects of alcohol on health outcomes 9. This approach was used recently by a study of Chinese individuals (*n* = 4200) in which a genetic marker in the alcohol-metabolizing gene, *ALDH2*, was used as an instrument for alcohol exposure 10; the study found no association between moderate alcohol intake and cognition in instrumental variable analysis. However, this study was relatively small, and limited to light and moderate drinking East Asian men. Larger studies, which include women and a greater range of alcohol intake, are needed to further investigate the inconsistency between observation and Mendelian randomization findings.

In populations of European descent *ALDH2* SNP rs671 is monomorphic, preventing its use in Mendelian randomization. However, a non-synonymous SNP (rs1229984) in the *ADH1B* gene, encoding one of the alcohol dehydrogenase family of enzymes (alcohol dehydrogenase 1B, *ADH1B*), is suitable for Mendelian randomization 11. Alcohol dehydrogenases are involved in metabolizing most of ingested alcohol 12,13. *In-vitro* studies have shown that A carriers of *ADH1B* rs1229984 have higher *ADH1B* enzymatic activity than G/G wild-type 14, and studies in humans have demonstrated that carriers of the A allele are less likely to drink alcohol and, if they do, have lower consumption than GG homozygotes 11,15. Several previous studies have used *ADH1B* rs1229984 in Mendelian randomization studies of various outcomes 16–18.

We investigated the association of rs1229984 with cognitive performance in six large epidemiological cohorts comprising more than 34 000 European participants, to obtain unbiased estimates of the association between alcohol consumption and cognitive function.

## Methods

### Ethics statement

All studies were approved by ethical committees in each participating centre and at UCL. All participants provided written consent to participate in the study.

### Study populations and study participants

These analyses combine data from the following large epidemiological cohorts.

#### English Longitudinal Study of Ageing (ELSA)

The ELSA sample was drawn from households that responded to the Health Survey for England (HSE) in 1998, 1999 and 2001. Households were included in ELSA if one or more resident was aged 50 years or more. There were 19 924 individuals in households that responded to HSE who would have been aged 50 by the time the ELSA sample was taken in 2002. Two thousand, five hundred and six of these older individuals died or were ineligible for follow-up; of the remainder, 11 392 (65.7%) became ELSA respondents. More detail has been reported elsewhere 19,20. Data used here are from the second wave of ELSA (2004), in which there were 7079 participants of a clinic visit; of these 5642 white/European participants provided DNA.

#### Whitehall II study

The Whitehall II cohort initially recruited 10 308 participants between 1985 and 1988 (Phase 1) from 20 London-based civil service departments 21. These participants were re-contacted between 1989 and 2004 on seven occasions. Data reported here are from Phase 7 (2002–04) of the Whitehall II study. Of 6941 participating at Phase 7, 6483 (93.4%) had a clinical assessment during which cognitive function assessments were administered. Of these, DNA is available from 5059 white/European participants.

#### Health, Alcohol and Psychosocial Factors In Eastern Europe (HAPIEE) study

The HAPIEE study recruited four random urban population samples of men and women aged 45–69 years at baseline in 2002–05 in Novosibirsk (Russia), Kaunas (Lithuania), Krakow (Poland) and six towns in the Czech Republic 22. Cognitive function was assessed in a subsample of participants at baseline and for the total sample at re-examination in 2006–08; during the re-examination, a fourth cohort in Kaunas (Lithuania) was established. A total of 36 030 people were recruited (overall response rate 61%), of whom 23 884 participants had data on both *ADH1B* rs1229984 genotype and cognition. Where participants had repeated measurements of cognitive function, the first measurement was used in the analysis.

### Measurements

In each study, participants completed questionnaires, underwent a clinical examination and provided blood samples.

#### Alcohol intake

Of the various aspects of alcohol consumption collected in each cohort, the measure comparable across cohorts was weekly consumption of alcohol. In ELSA and Whitehall II, the question related to intake during the last week (reported in British units) and in the HAPIEE cohorts, to a typical week (reported in litres and millilitres). For the purposes of this analysis, we converted all values to British units (1 British unit = 8 g of ethanol). Given the relatively small proportion of participants who reported consuming more than the recommended amounts (28 units per week in men and 21 units in women), drinkers in our cohorts largely represent light-to-moderate drinkers.

#### Cognitive performance

Memory was assessed by immediate recall (all cohorts) and delayed recall (ELSA and HAPIEE cohorts). In ELSA, 10 common words were read out by a computer at a rate of one word every 2 seconds. The sound level was adjusted to meet the requirements of each participant. Participants were asked to recall as many words as possible immediately and again after a short delay, during which they completed other cognitive tests. Four different randomly assigned word lists were used, and members of the same household were given different versions. Whitehall II participants were given 1 minute to write down the number of words remembered. In the HAPIEE cohorts, a list of the same 10 words was read by an interviewer (baseline) or from a computer (re-examination) three times. Participants were asked to recall verbally as many words as possible immediately and then again after completing all other cognitive tests. The sum of the three immediate recalls was used in the analysis.

Verbal (semantic) fluency was assessed in all cohorts by recalling as many animals as possible in 1 minute. In HAPIEE and ELSA cohorts recall was verbal and results were recorded by the interviewer, while in Whitehall II, participants were asked to write down their responses.

Processing speed was assessed in ELSA and HAPIEE using the letter cancellation test. Participants were asked to cross out as many of the 65 target letters (‘*P*’ and ‘W’, ‘*P*’ and ‘III’ in Russia) as possible in 1 minute on a page incorporating 780 letters in a grid. The total number of correctly letters crossed-out letters was used as a measure of processing speed.

### Genotyping and quality control

The rs1229984 variant in *ADH1B* was genotyped in all six cohorts. Genotyping was carried out using the Kaspar genotyping platform by KBioscience (http://www.lgcgenomics.com) in Russian, Polish and Lithuanian HAPIEE samples and in ELSA and Whitehall II studies. Czech HAPIEE samples were genotyped at the Institute of Clinical and Experimental Medicine, Prague. A subsample of 100 samples was genotyped at both laboratories, with 99% agreement. In all cohorts, the call rate was >98% and the rs1229984 SNP was in Hardy–Weinberg equilibrium at *P* > 0.001 (Table 1).

**Table 1:** Descriptive characteristics of the analytical samples

	HAPIEE Czech Republic	HAPIEE Russia	HAPIEE Poland	HAPIEE Lithuania	Whitehall II	ELSA
No. subjects	5607	5814	5627	6836	5031	5537
Age, mean, SD, years	59.1 (7.0)	59.7 (6.9)	58.8 (7.0)	60.9 (7.6)	55.3 (6.0)	66.1 (9.8)
% women	55	57	51	54	26	54
Cognitive function (mean, SD)						
Word recall (max. 30)^a^	22.6 (3.6)	20.9 (4.6)	20.4 (4.3)	21.8 (4.1)	7.1 (2.4)	5.7 (1.9)
Delayed recall (max. 10)	7.6 (1.8)	7.0 (2.2)	7.1 (1.9)	7.7 (1.9)	NA	4.4 (2.1)
Verbal fluency	23.6 (6.6)	18.6 (7.1)	21.0 (6.3)	21.4 (6.2)	16.95 (4.0)	20.2 (6.5)
Letter search	17.9 (4.7)	17.0 (5.4)	18.0 (5.8)	16.2 (4.8)	NA	10.4 (3.6)
Weekly alcohol intake, *n* (median, IQR)	5491 (5, 18)	5813 (0, 3)	5556 (0, 6)	6812 (2, 6)	4644 (10, 17)	4587 (2, 12)
*ADH1B* genotype, *n* (%)						
GG	5024 (89.6)	5203 (89.5)	5055 (89.8)	6375 (93.3)	4758 (94.6)	5235 (94.6)
AG	568 (10.1)	601 (10.3)	552 (9.8)	457 (6.7)	266 (5.3)	291 (5.3)
AA	15 (0.3)	10 (0.2)	20 (0.4)	4 (0.1)	7 (0.1)	11 (0.02)
HWE *P*-value	*P* = 0.90	*P* = 0.09	*P* = 0.23	*P* = 0.20	*P* = 0.11	*P* = 0.004
Call rate (%)	99.6	98.7	98.8	98.9	99.3	98.8

a Word recall was based on 30 words in the Health, Alcohol and Psychosocial factors In Eastern Europe (HAPIEE) study and 10 words in the Whitehall II study. ELSA = English Longitudinal Study of Ageing; HWE = Hardy–Weinberg equilibrium; IQR = interquartile range; NA = not available; SD = standard deviation; *AD1HB* = alcohol dehydrogenase 1B.

### Confounders and mediators

Sex and age were obtained from questionnaire information in each of the cohorts. Age was used in analysis in 5-year age-bands. Educational attainment, which is considered as a mediator in the association of alcohol intake and cognitive performance, is categorized as attainment of primary, vocational, secondary and university/postgraduate education.

### Statistical analyses

All cognitive test results were standardized (mean = 0, standard deviation = 1) to allow comparison between tests. A dominant model was used for genetic analyses; that is, AA carriers and GA carriers were compared to the GG reference group. Owing to a skewed distribution, alcohol intake in units per week was natural log-transformed. A value of 1 was added prior to log-transformation in order to preserve values for individuals who consumed no alcohol.

We generated a pooled data set for the analyses and adjusted for study cohort. First, we examined the observational association between alcohol and cognitive traits. To do this, we generated categories of alcohol consumption (0, >0–<5, ≥5–<10, ≥10–<15, ≥15–<20, ≥20 British units/week) and conducted linear regression analyses with the cognition traits as dependent variables and alcohol categories as the independent variable, adjusted for age group (used in 5-year age-bands) and sex. Individuals reporting 0 units/week alcohol were used as the reference group.

Secondly, we investigated the association of the gene variant on any versus no alcohol and on alcohol intake in log weekly units per week. Because a Mendelian randomization analysis is conducted in part to remove potential confounders, we also investigated the association between the rs1229984 A-allele and common confounding factors, such as age, sex and smoking. Educational attainment was considered a potential mediator in the association of rs1229984 A-allele with cognition.

We investigated the association of the rs1229984 A-allele with cognitive traits overall and separately for those who reported no drinking versus those who report alcohol intake. We conducted a test for interaction between *ADH1B* rs1229984 and each cognitive trait stratified by any versus no alcohol consumption under the hypothesis that the *ADH1B* rs1229984 variant would only act in individuals exposed to alcohol; a small *P*-value for interaction would provide evidence of a differential effect of the genetic variant according to alcohol consumption.

Thirdly, we used the gene variant to estimate the unconfounded associations between alcohol and cognitive scores. We used the observational association to inform on the approach for the instrumental variable analysis. The observational associations suggested a plateau effect, where any alcohol consumption per week was associated with higher cognitive scores compared with individuals consuming 0 units per week, with no apparent gradient among drinkers. We dichotomized the alcohol exposure into any versus none per week, and used the gene variant to instrument this alcohol phenotype. The instrumental variable estimates therefore relate to the effect of any alcohol compared with none per week on cognitive function. In sensitivity analysis, we used the genetic variant to instrument volume of alcohol in log-weekly units. We used two-stage least-squares instrumental variable analysis using the ‘ivregress’ command in Stata version 12.0 (StataCorp, College Station, TX, USA). The analyses were repeated after excluding individuals consuming more than the recommended limit to examine light-to-moderate drinking.

Finally, to maximize use of the available data, we combined our findings with those from a recently published study using the *ALDH2* gene 10. We generated instrumental variables estimates for a 1-unit increase in alcohol per day and its effect on delayed recall in our pooled data set. Using this instrumental variable estimate, we conducted a fixed-effects meta-analysis using the previously published instrumental variable estimate for a comparable difference in alcohol on a delayed 10-word recall trait.

## Results

### General characteristics

There were 34 452 individuals in the analysis, with a mean age ranging from 44 to 66 years; 50% of participants were female. The median alcohol consumption in the pooled data set was 2.3 units per week [interquartile range (IQR) 0, 11.4]. No major differences in cognitive test scores among the six cohorts were observed; the lower scores in ELSA reflected the older age of participants in this cohort (Table 1).

### Observational associations between alcohol and cognition traits

**Figure 1:**
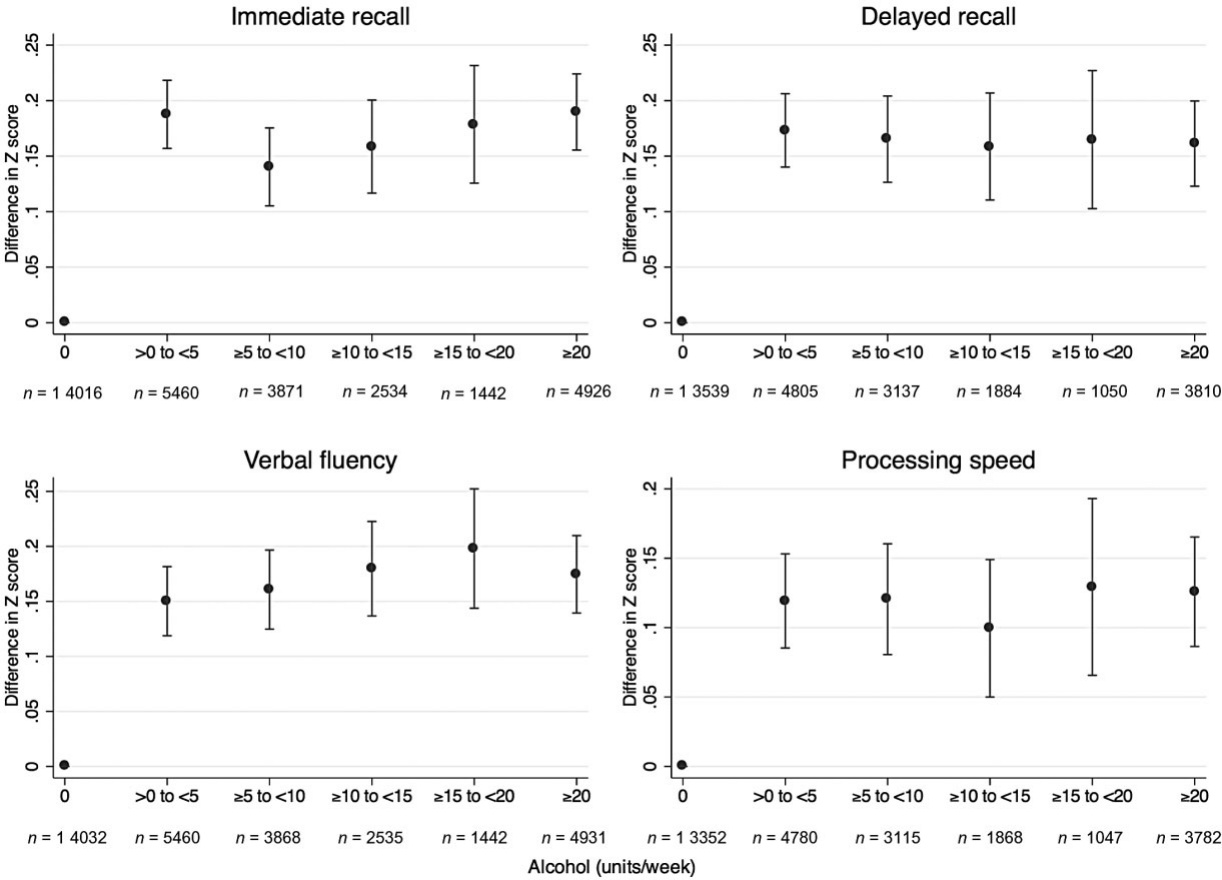
Observational association between alcohol intake (categories of volume compared to no alcohol) and cognitive traits. Estimates are adjusted for age group and sex. Blue dots represent the mean and red whiskers represent the 95% confidence interval

Scores for all four cognitive traits were higher in individuals who consumed any alcohol compared to individuals who consumed no alcohol per week, with a ‘plateau’ effect whereby the greatest difference in cognitive function was between individuals who reported drinking 0 units per week and individuals who consumed between >0 to <5 units per week, and no difference within individuals who consumed any alcohol per week (Fig. 1).

### Association of *ADH1B* rs1229984 with alcohol intake

Carriage of the A-allele of rs1229984 was associated with a 10.3% lower alcohol intake per week [95% confidence interval (CI) = 5.6%, 14.7%) with an *F*-statistic of 41 and *R*^2^ of 0.1%. The corresponding odds ratio for any versus no weekly alcohol was 0.87 (95% CI = 0.80, 0.95) (Table 2) with *F*-statistic of 47 and *R*^2^ = 0.1%.

**Table 2:** Difference in alcohol phenotypes, age, sex, smoking status and educational attainment between the *ADH1B* rs1229984 A-allele carriers versus GG homozygotes (reference group)

Trait	No. with a trait/total	Effect estimate (95% CI)	P-value
Alcohol traits			
Units/week	NA/32 903	−10.3% (−14.7%, −5.6%)^a^	3.1 × 10^−5^
Any versus no alcohol intake per week	18 778/32 903	0.87 (0.80, 0.95)^b^	0.001
Confounders			
Age group, years (<50, ≥50–<55, ≥55–<60, ≥60–<65, ≥65–<70, ≥70–<75, ≥75–<80. ≥80–<85, ≥85–<90, ≥90)	NA/34 316	1.01 (0.95 1.09)^c^	0.71
Sex (male)	17 116/34 431	1.10 (1.01, 1.19)^b^	0.024
Smoking status (ever versus none)	16 671/33 897	1.080 (0.998, 1.170)^b^	0.06
Potential mediator			
Educational attainment (primary, vocational, secondary, university)	NA/33 726	1.14 (1.06, 1.22)^c^	3.0 × 10^−4^

a Linear regression coefficient;

b odds ratio;

c estimates obtained from ordered logistic regression, therefore the odds ratio refers to the odds of increasing from one category to the next higher. CI = confidence interval; NA = not available.

### Association of *ADH1B* rs1229984 with age, sex, smoking and educational attainment

No association was found between carriage of the A-allele of rs1229984 with age. However, a weak association was identified with male sex [odds ratio (OR) = 1.10; 95% CI = 1.01, 1.19; *P* = 0.02) and smoking status (OR = 1.08; 95% CI = 1.00–1.17; *P* = 0.06) while the association with educational attainment was significant (OR = 1.14; 95% CI = 1.06, 1.22 for each unit increase in educational category; *P* = 3 × 10^−4^, Table 2).

### Association of *ADH1B* rs1229984 with cognitive traits overall and stratified by alcohol consumption

We found no association between carriage of the A-allele of rs1229984 and cognitive performance, neither overall nor when stratified by drinking status (into drinking any versus no alcohol per week) (all *P*-values >0.1; Table 3). Using the genotype as a continuous variable produced similar results. The interaction between alcohol intake and the rs1229984 A-allele carrier status was not significant (all *P*-values >0.1), meaning that there was no differential association between the rs1229984 A-allele and cognition when stratified by alcohol consumption.

**Table 3:** Association of the alcohol dehydrogenase 1B (*ADH1B*) rs1229984 A-allele carriage^a^ (versus GG reference group) with cognitive performance, stratified by alcohol intake

Cognitive test	Alcohol stratum	Number	Regression coefficient (95% CI)	P-value	P-value (interaction)^b^
Immediate recall	All individuals	33 512	0.02 (−0.02, 0.06)	0.40	NA
	No alcohol	14 016	0.03 (−0.03, 0.09)	0.31	0.35
	Any alcohol	18 233	0.01 (−0.05, 0.07)	0.72	
Delayed recall	All individuals	29 386	0.03 (−0.02, 0.07)	0.20	NA
	No alcohol	13 539	0.03 (−0.03, 0.09)	0.30	0.53
	Any alcohol	14 686	0.03 (−0.03, 0.09)	0.36	
Verbal fluency	All individuals	33 531	0.01 (−0.03, 0.05)	0.47	NA
	No alcohol	14 032	0.02 (−0.04, 0.07)	0.54	0.95
	Any alcohol	18 236	0.03 (−0.02, 0.09)	0.23	
Processing speed	All individuals	29 105	−0.01 (−0.05, 0.03)	0.68	NA
	No alcohol	13 352	−0.02 (−0.08, 0.04)	0.56	0.60
	Any alcohol	14 592	0.01 (−0.05, 0.07)	0.68	

a A-allele carriage is associated with reduced alcohol consumption.

b Likelihood ratio test for interaction between alcohol intake (any versus none per week) and *ADH1B* rs1229984 (A carriage versus GG, reference group) on cognitive performance. Alcohol intake (no alcohol, any alcohol) refers to consumption per week. The ‘All individuals’ stratum includes participants without measures of alcohol intake. CI = confidence interval; NA = not available.

### Contrasting observational to instrumental variable analyses

Because the observational association between alcohol intake and cognition suggested that the greatest difference in cognitive traits was when comparing any alcohol intake to none per week, we used the rs1229984 variant to instrument this phenotype. The observational associations suggest that alcohol intake was associated consistently with higher cognitive scores (Table 4). In contrast, the instrumental variable estimates for any versus no alcohol intake per week showed no association between alcohol intake and cognitive traits. Further, the point estimates from these analyses were directionally opposite to those of the observational estimates, although the 95% CI of the instrumental variable estimates were wide and overlapped those of the observational estimates. Similar results were apparent when analyses were repeated using the effect of a 1-log unit increase in alcohol intake per week (Table 4). We additionally adjusted for educational attainment and smoking status, and found the instrumental variable estimates to remain unaltered (Table 4). With the exception of delayed recall, *P*-values for the Durbin–Wu–Hausman test were >0.05, suggesting no difference between the observational and instrumental variable estimates. Repeating the instrumental variable analyses with participants consuming fewer than recommended drinking limits did not change the results (not shown).

**Table 4:** Observational and instrumental variable estimates for the association between cognitive phenotype and alcohol intake: any versus no alcohol per week (upper part) and for a 1-log unit increase in alcohol consumption per week (lower part)

	Immediate recall	Delayed recall	Verbal fluency	Processing speed
Any drinking versus none				
Observational (1)	0.17 (0.15, 0.20)	0.17 (0.14, 0.19)	0.17 (0.14, 0.19)	0.12 (0.09, 0.15)
Instrumental variable (1)	−0.74 (−1.88, 0.41)	−1.09 (−2.38, 0.21)	−0.63 (−1.78, 0.53)	−0.16 (−1.29, 0.97)
*P*-value (Durbin–Wu–Hausman test)	0.09	0.03	0.15	0.63
Instrumental variable (2)	−0.23 (−1.17, 0.72)	−0.60 (−1.62, 0.42)	−0.16 (−1.13. 0.81)	0.21 (−0.77, 1.19)
*P*-value (Durbin–Wu–Hausman test)	0.48	0.15	0.61	0.78
For 1-log unit increase in alcohol consumption per week				
Observational (1)	0.05 (0.04, 0.06)	0.05 (0.04, 0.06)	0.05 (0.04, 0.06)	0.04 (0.03, 0.05)
Instrumental variable (1)	−0.20 (−0.50, 0.10)	−0.33 (−0.71, 0.05)	−0.17 (−0.48, 0.14)	−0.05 (−0.40, 0.30)
*P*-value (Durbin–Wu–Hausman test)	0.09	0.03	0.14	0.63
Instrumental variable (2)	−0.06 (−0.32, 0.20)	−0.19 (−0.51, 0.13)	−0.04 (−0.31, 0.22)	0.07 (−0.24, 0.38)
*P*-value (Durbin–Wu–Hausman test)	0.48	0.16	0.60	0.77

Estimates are regression coefficients (95% confidence interval) for drinking any alcohol versus no alcohol per week (upper part) and for a 1-log unit increase in alcohol volume per week (lower part). (1) Adjusted for age group and sex; (2) adjusted for age, sex, smoking and education. *P*-value (Durbin–Wu–Hausman test) represents a test for endogeneity; a small *P*-value can be interpreted that the observational and instrumental variable estimate are non-concordant.

**Figure 2:**
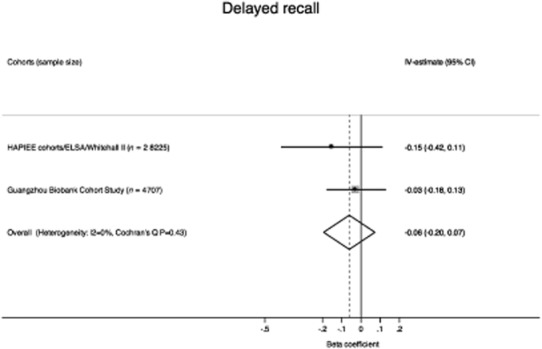
Meta-analysis of instrumental variable estimates to investigate the totality of available evidence on the association of alcohol with delayed recall. Instrumental variable (IV) estimates are for a 1-unit increase in alcohol per day. Although the Guangzhou Biobank Cohort Study had fewer participants, the gene used had a stronger effect on alcohol consumption

To maximize use of the available data, we combined our data with previously published 10 causal estimates for a 1 unit/day increase in alcohol on delayed recall using fixed-effects meta-analysis. The combined analysis, including 32 932 participants, found that a 1-unit/day increase in alcohol did not associate with delayed recall (instrumental variable beta coefficient for a 1-unit increase in alcohol/day: −0.06; 95% CI = −0.20, 0.07), with low heterogeneity between estimates (*I*^2^ = 0%) (Fig. 2).

## Discussion

In data from six large population-based cohorts, we found strong evidence of alcohol intake associated with better cognitive performance. However, a Mendelian randomization analysis, using a genetic variant (*ADH1B* rs1229984) associated strongly with alcohol intake, did not replicate the observational estimate, although the confidence intervals were wide due to the genetic instrument explaining only a small proportion of variance of alcohol intake. Given that the instrumental variable analysis findings are less likely to be biased than the observational estimates, our findings do not support the hypothesis that alcohol consumption has a beneficial effect on cognitive function.

The main advantage of Mendelian randomization analysis is that it minimizes bias and confounding that can hamper observational studies. Although we found a weak association between the *ADH1B* rs1229984 variant with sex, this association is most probably a false positive; in addition, we did not identify differential effects of the genetic variant on cognitive traits in sex-stratified analyses (data not shown) and we adjusted for sex in the instrumental variable analysis. The association of the rs1229984 variant with educational attainment may reflect an inverse association of alcohol consumption in young adulthood with educational attainment, but could also reflect parental alcohol intake 23. We speculate that educational attainment in early life could be on a causal pathway between alcohol intake and cognition, given the extensive evidence that education is related causally to cognitive performance 24,25. However, despite the association of the genetic variant with educational attainment and a strong association of education with cognition in our data, the genetic variant did not associate with cognition in our analyses.

Our study has several benefits. This is the largest study conducted thus far to investigate the role of alcohol on cognition through Mendelian randomization. The participating cohorts covered a wide range of societal contexts with a large variation of drinking patterns and pronounced differences in health. Although this introduces heterogeneity between the studies, it also allows us to generalize the association of alcohol intake and cognitive performance, which was examined previously only in a low-consumption East Asian population using the Mendelian randomization approach 10. We were also able to conduct analyses in a pooled data set in which the alcohol and cognitive traits were harmonized between studies.

Several limitations should be considered when interpreting these results. First, while our study contains data from more than 34 000 participants, very large numbers of observations are required for an instrumental variable analysis using a single nucleotide polymorphism (SNP) that explains only 0.1% of the *R*^2^ of alcohol. Our Mendelian randomization estimates are therefore less precise than those of the observational estimates, and the statistical power to show inverse association with cognitive functions was approximately 10%. Our results should therefore be replicated with even larger sample sizes (or through using multiple SNPs in combination to increase the variance explained in alcohol consumption). While our study was almost 10-fold larger than the one previous Mendelian randomization study to have addressed this question 9, our genetic instrument was weaker and our estimates were therefore less precise. When we incorporated the published findings with ours similar findings were obtained, showing a consistent null effect of alcohol on delayed recall. Further, a recent study in 3542 men failed to observe an association of association of *ADH1B* with cognitive impairment or decline in older age groups 26. Despite this, we still cannot exclude the possibility of a false-negative finding.

Secondly, we used cross-sectional data, rather than decline in cognitive performance over time. This would be a severe limitation of a traditional observational study, as people change their drinking habits over time and drinkers often stop drinking because of health or other problems. However, the Mendelian randomization design reduces this problem by using a genetic marker of long-term exposure to alcohol intake.

Thirdly, the four measures of cognition used in these analyses were limited to those available in the six cohorts and did not allow investigation of clinically meaningful cognitive outcomes such as dementia. Nevertheless, cognitive test performance predicts future cognitive impairment and dementia 27, which are proposed to be the result of long-term processes that occur over decades 28,29. As genetic variants are determined at meiosis and are related to life-long differences between individuals, Mendelian randomization is particularly pertinent to questions concerning effects on conditions with a long pre-clinical phase, such as cognitive impairment and cardiovascular disease 18, to minimize bias from reverse causality.

Fourthly, a possible limitation of Mendelian randomization studies is a potential association of an indirect variant (*ADH1B* in our study) with non-endogenous exposures. However, *ADH1B* encodes the enzyme alcohol dehydrogenase 1B, involved in the primary metabolic pathway of alcohol 30. It is therefore very unlikely that *ADH1B* would directly influence cognition other than through alcohol.

Fifthly, we used a linear instrumental variable analysis approach, which might seem at odds with the observational estimates we identified as a non-monotonic increase in cognitive function with alcohol. For this reason, we dichotomized alcohol into any versus none per week, allowing us to mimic the observational relationship for the instrumental variable analysis. Furthermore, as previous observational studies link better cognitive function with light-to-moderate drinking, we conducted additional analyses excluding participants who drink more than the recommended levels, but the results remained unaltered.

Finally, population stratification can compromise Mendelian randomization analysis 31; however, as our analyses were restricted to white Europeans with similar allele frequencies, this is unlikely to be a problem. Furthermore, adjustment for principal components in the two cohorts in which this was feasible did not alter the estimate (data available on request).

We conclude that using a Mendelian randomization approach in a large data set of more than 34 000 individuals in the general population did not identify strong evidence of a causal relationship between alcohol intake and cognitive function, due to limited power. Although this suggests that the observational estimates may be influenced by bias or confounding, almost 1 million participants would be required for a conclusive Mendelian randomization study using *ADH1B*. Therefore, further Mendelian randomization studies in larger samples and/or using stronger genetic instruments are required to replicate these findings.
